# Quincke Triad and Hepatic Artery Pseudoaneurysm Presenting to the Emergency Department: A Case Report

**DOI:** 10.5811/cpcem.35484

**Published:** 2025-04-01

**Authors:** Courtney Wham, Daria Nicke, Justin Burman, Robert Meller, Premal Trivedi, Andra Farcas

**Affiliations:** * Denver Health, Emergency Medicine Residency, Denver, Colorado Denver Health; † Paramedic Division, Denver, Colorado; ‡ University of Colorado School of Medicine, Department of Vascular and Interventional Radiology, Aurora, Colorado; § University of Colorado School of Medicine, Department of Emergency Medicine, Aurora, Colorado

**Keywords:** hepatic artery pseudoaneurysm, endovascular repair

## Abstract

**Introduction:**

Hepatic artery aneurysms are exceedingly rare, often asymptomatic, and usually diagnosed when patients present with complications such as rupture or bile duct obstruction.

**Case Report:**

This report describes a 70-year-old female who presented to the emergency department with Quincke triad (epigastric pain, obstructive jaundice, and gastrointestinal bleeding) and was diagnosed with multiple hepatic artery pseudoaneurysms with a thrombosed fistulous connection to the biliary system. She was treated effectively with extensive embolization and biliary stenting.

**Conclusion:**

This case underscores the importance of early diagnosis and highlights the role of multidisciplinary intervention in preventing life-threatening complications from hepatic artery aneurysms.

## INTRODUCTION

Hepatic artery aneurysms (HAA) occur in 0.002–0.4% of the population and account for 14–20% of all visceral artery aneurysms.^1^,^2^ Most HAAs are asymptomatic, with 60–80% of patients not diagnosed until complications such as rupture, bleeding, or obstructive jaundice from bile duct compression arise.^3^ The most common presentation is Quincke triad (epigastric pain, obstructive jaundice, and gastrointestinal [GI] bleeding), which is present in approximately 56% of cases.^4^ Here we report a rare case of an elderly female patient presenting with Quincke triad who was found to have complex common, proper, and right hepatic artery pseudoaneurysms with a thrombosed fistulous connection to the biliary tree. Pseudoaneurysms are collections of blood that form outside the blood vessel but are contained by connective tissue, whereas true aneurysms are permanent bulges that form in all three layers of the vessel. This pseudoaneurysm was managed effectively with extensive embolization and biliary stenting.

## CASE REPORT

A 70-year-old female with a remote history of a cholecystectomy (performed over 14 years prior) presented to the emergency department (ED) via ambulance for hematemesis. She had experienced a week of intermittent nausea and vomiting, culminating in a single episode of frank red blood in her emesis. Emergency medical services reported hypotension in the field, with a systolic blood pressure of 60 millimeters of mercury (mm Hg), and bradycardia ranging between 50–60 beats per minute. Following administration of one liter of normal saline in the prehospital setting, her systolic blood pressure improved to 100 mm Hg.

**Image 1:**
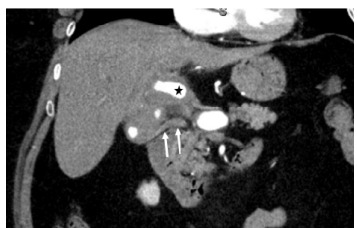
Coronal image of abdominal computed tomography with angiography showing a hepatic artery aneurysm (star) with an associated high-density common bile duct (arrows), indicating thrombosis in the biliary system.

Upon arrival at the ED, the patient had blood pressure of 102/69 mm Hg, a heart rate of 59 beats per minute, a respiratory rate of 18 breaths per minute, a temperature of 36.3 °Celsius, and room air oxygen saturation of 97%. Examination revealed dried blood in the oropharynx, scleral icterus, jaundice, and tenderness in the epigastric region. Initial laboratory results showed a mild leukocytosis of 11,490/milliliter (mL) (reference range: 4,000–11,100/mL), a hemoglobin level within normal limits at 14.0 gram (g) per deciliter (dL) (12.1–16.3 g/dL), an elevated serum creatinine of 1.14 milligrams (mg) per dL (0.60–1.20 mg/dL) with a baseline of 0.8 mg/dL from two months prior, and significantly increased liver enzymes with a total bilirubin of 5.4 mg/dL (0.1–1.3 mg/dL), an alkaline phosphatase of 464 international units (IU)/liter (L) (39–117 IU/L), an alanine aminotransferase of 1,152 IU/L (7–52 IU/L), and an aspartate aminotransferase of 507 IU/L (12–39 IU/L). Computed tomography with angiography (CTA) of the abdomen and pelvis identified a 3.1-centimeter saccular pseudoaneurysm of the extrahepatic artery with intraluminal thrombus and severe stenosis of the hepatic artery (Image 1). Mild intrahepatic biliary ductal dilation suggested biliary obstruction, likely due to compression. The CTA did not identify a clear source of the GI bleeding.

A transfer was requested to a facility with endovascular capabilities for definitive management. Before transfer, the patient had one episode of hematochezia but remained hemodynamically stable. Prior to departure, she received 4 mg of intravenous (IV) ondansetron, 80 mg of IV pantoprazole, and a liter of lactated Ringer. Upon arrival at the receiving facility, her clinical condition remained unchanged. General surgery, interventional radiology (IR), and gastroenterology were consulted, and she was promptly taken to the IR suite for embolization.

Gastroenterology was consulted regarding the administration of ceftriaxone and octreotide given biliary pathology with concern for GI bleeding but ultimately recommended against these medications, as there was low concern for esophageal varices given abrupt onset of jaundice and absence of risk factors such as cirrhosis. The general surgery team recommended IR and GI intervention and, if unsuccessful, they suggested hepatobiliary surgical consultation to evaluate for liver transplant.

Interventional radiology review of CTA imaging revealed extensive aneurysm extending from the common hepatic artery to the right hepatic artery deep into the hepatic hilum with the proper and right hepatic artery components partially thrombosed. Additionally, the patient was noted to have some intrahepatic biliary dilation, and the common bile duct appeared to be of higher density than usual, suggesting presence of blood within it (Image 1).

**Image 2:**
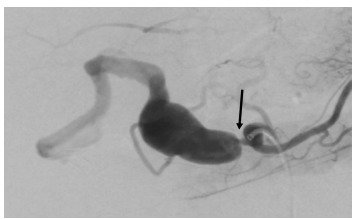
Complete angiography demonstrating tight stenosis of the proximal common hepatic artery (arrow).

The patient’s GI bleeding was attributed to a suspected erosion of a HAA into the biliary system, forming a fistulous connection. Due to the presence of a large aneurysm, the patient was taken directly for angiography and embolization. Selective angiography of the vessels revealed an irregular aneurysmal celiac trunk with high-grade irregular stenosis of the common hepatic artery ostium (Image 2).

**CPC-EM Capsule**

What do we already know about this clinical entity?

*Hepatic artery aneurysms are rare and often asymptomatic, but they can present with life-threatening complications such as rupture, bleeding, or biliary obstruction*.

What makes this presentation of disease reportable?

*This case highlights a rare case of hepatic artery pseudoaneurysms with a thrombosed arterial-biliary fistula that was managed effectively with embolization and stenting*.

What is the major learning point?

*Early recognition of hepatic artery aneurysm is critical and often lifesaving and requires prompt identification and multidisciplinary intervention*.

How might this improve emergency medicine practice?

*Maintain high suspicion for hepatic aneurysms in gastrointestinal bleeding with painful jaundice, especially with a history of prior cholecystectomy*.

**Image 3:**
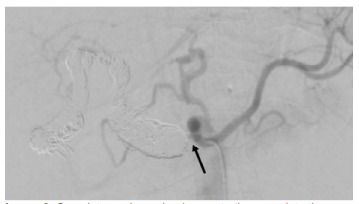
Complete angiography demonstrating complete thrombosis of the extensive aneurysmal hepatic artery (arrow indicating inception point) with preserved arterial flow in the splenic artery and gastrohepatic artery.

There was also extensive aneurysmal common hepatic, proper hepatic, and right hepatic arteries with intervening areas of stenosis. Additionally, there was irregular outpouching of the distal right hepatic artery adjacent to the cholecystectomy clips, possibly representing thrombosed fistula to the biliary tree (Image 3).

During embolization, over 60 coils were deployed to achieve hemostasis. At the conclusion of the procedure, there was complete thrombosis of the extensive aneurysmal common hepatic, proper hepatic, and right hepatic arteries. Notably, arterial flow to the left hepatic lobe was preserved due to variant origin from the left gastrohepatic trunk and, after embolization, supply to the right hepatic lobe was also preserved through left-to-right intrahepatic shunting. There were no immediate operative complications.

On hospital day two, the patient underwent an endoscopic retrograde cholangiopancreatography (ERCP). Contrast revealed diffuse dilation of the hepatic and intrahepatic ducts upstream of a stenosis. Filling defects in the bile ducts, believed to be sludge, were identified, and a balloon was used to sweep clots and old blood from the ducts, confirming the original diagnosis. A biliary sphincterotomy was performed, and a temporary, covered metal biliary stent was placed to address the narrowing in the common hepatic duct. Excellent flow was observed after stent placement.

The patient was discharged on hospital day three with down-trending liver enzymes, improving serum creatinine, and plans for a repeat ERCP in two months.

## DISCUSSION

Hepatic artery aneurysms and pseudoaneurysms are infrequent, accounting for a fraction of all visceral artery aneurysms, and are often asymptomatic until they present with complications such as rupture or bile duct obstruction.^1^,^2^ The reported mortality of a ruptured HAA is 80–100%, making its prompt recognition and treatment critical to patient survival.^1^ This case highlights the importance of considering an arterial-biliary fistula from a HAA when patients present with GI bleeding in the absence of other clear causes.

True aneurysms are usually found as a single extrahepatic lesion, whereas pseudoaneurysms are normally found as multiple intrahepatic lesions, such as in this case.^4^ While the terms aneurysm and pseudoaneurysm were used interchangeably and variably among specialists on the case, the patient’s remote history of a laparoscopic cholecystectomy was her strongest risk factor for the development of pseudoaneurysms. Albeit incredibly rare with the incidence of vascular complications after a cholecystectomy at 0.3%, a recent review of 135 cases of hepatic pseudoaneurysms found that 28% of all cases were associated with prior laparoscopic cholecystectomies.^5^ This makes hepatic artery pseudoaneurysm a diagnosis to consider on the painful jaundice differential list, especially in a patient with a history of a laparoscopic cholecystectomy. This same study found that 31% of hepatic pseudoaneurysms were secondary to a history of percutaneous transhepatic biliary drainage, and 13% were associated with a history of an open cholecystectomy.^5^ The presence of these risk factors in a patient’s history should add hepatic artery pseudoaneurysm to the differential list, although the rarity of the condition puts it lower.

Although an appropriate initial imaging evaluation of painful jaundice is an abdominal ultrasound per the American College of Radiology, the rare nature of this condition has not provided enough data for calculation of specificity and sensitivity of this modality in the diagnosis of HAA.^6^ The gold standard imaging to identify a HAA is digital subtraction angiography, as determined in a subset of liver transplant patients for whom hepatic aneurysms are more common postoperatively.^7^ However, this is not likely to be a viable option for diagnosis in most EDs. Another study found that hepatic arterial complications in a subset of liver transplantation patients diagnosed by ultrasound are often nonspecific and need further categorization with computed tomography imaging.^8^ However, due to the infrequency of HAAs, specific guidelines are not present. Notably, CTA has excellent diagnostic accuracy for detecting HAAs with a sensitivity and specificity of 100%. Given the availability of this imaging modality the ED, it should be prioritized in patients with concern for abdominal aneurysms.^3^ While a right upper quadrant ultrasound in the stable patient presenting with obstructive jaundice may be an appropriate starting point, clinicians should have a low threshold to escalate to CTA if the cause of the jaundice is not explained by ultrasound findings and especially if it is accompanied by any GI bleeding.

Once an HAA is identified, there is a lot of variability in the treatment modalities due to the infrequency of the condition and the publication of only single-case studies or metanalyses of case studies to guide treatment. Guidelines from the Society of Vascular Surgery recommend an endovascular-first approach to all hepatic aneurysms and pseudoaneurysms, and endovascular embolization is the most reported approach. Roughly 37% of all reported cases of HAA used coil embolization for treatment.^9^ Alternative treatment options are higher risk up to the need for liver transplantation.

While this patient remained hemodynamically stable during her ED and inpatient courses, the potential for instability underscores the importance of identification of this vascular pathology and proper consultation and transfer to a facility with IR. Case reports of patients who do present with hemorrhagic shock due to HAA rupture offer additional stabilization modalities, including one successful case using a resuscitative endovascular balloon occlusion of the aorta.^10^ Other treatment options include exploratory laparotomy with excision, ligation, and repair with arterial grafting and hepatic resections.^12^ Getting surgical and interventional radiological specialists on board early is paramount to patient survival in the event of HAA rupture.

## CONCLUSION

This case highlights the rarity and clinical complexity of hepatic artery pseudoaneurysms, particularly in the context of biliary complications. The patient’s presentation with Quincke triad and the subsequent discovery of multiple aneurysms and a thrombosed fistula underscore the importance of early recognition and multidisciplinary intervention in preventing life-threatening outcomes. The successful management through extensive embolization and biliary stenting, preserving hepatic function, emphasizes the critical role of getting interventional radiology and gastroenterology on board early. This report further advocates for heightened clinical suspicion of hepatic artery pseudoaneurysms, especially in patients with a history of laparoscopic cholecystectomy.
